# Death of Dr. Samuel Logan

**Published:** 1893-02

**Authors:** 


**Death of Dr. Samuel Logan.**—Dr. Samuel Logan, Professor of Surgery in Tulane University, New Orleans, died January 12, 1893, at the age of 63 years. He graduated in medicine in 1853, at the South Carolina Medical College. He served during the war as Surgeon and Medical Inspector under General Lee. He was connected with the South Carolina Medical College before and for a short time after the war, in the chair of anatomy and surgery. In 1866, he was elected to the chair of anatomy in the Medical College of Virginia, in Richmond, and removed to the latter city from Charleston. In 1867, he accepted the chair of surgery in the New Orleans School of Medicine, and in 1872 was elected professor of anatomy and clinical surgery in the University of Louisiana (Tulane). Dr. Logan was a prominent member and officer in a large number of medical and scientific associations; was regarded as one of the brightest teachers of medicine in the South; and his death leaves a vacancy not only in the teaching force of the school to which he was attached, but in the hearts of those who knew him.

